# Magnetic Flux Distribution of Linear Machines with Novel Three-Dimensional Hybrid Magnet Arrays

**DOI:** 10.3390/s17112662

**Published:** 2017-11-18

**Authors:** Nan Yao, Liang Yan, Tianyi Wang, Shaoping Wang

**Affiliations:** School of Automation Science and Electrical Engineering, Beihang University, Beijing 100191, China; yaonan@buaa.edu.cn (N.Y.); wangtianyi@buaa.edu.cn (T.W.); shaopingwang@vip.sina.com (S.W.)

**Keywords:** linear machine, hybrid magnet arrays, multiple movers, magnet flux, magnetic vector potential, FEM

## Abstract

The objective of this paper is to propose a novel tubular linear machine with hybrid permanent magnet arrays and multiple movers, which could be employed for either actuation or sensing technology. The hybrid magnet array produces flux distribution on both sides of windings, and thus helps to increase the signal strength in the windings. The multiple movers are important for airspace technology, because they can improve the system’s redundancy and reliability. The proposed design concept is presented, and the governing equations are obtained based on source free property and Maxwell equations. The magnetic field distribution in the linear machine is thus analytically formulated by using Bessel functions and harmonic expansion of magnetization vector. Numerical simulation is then conducted to validate the analytical solutions of the magnetic flux field. It is proved that the analytical model agrees with the numerical results well. Therefore, it can be utilized for the formulation of signal or force output subsequently, depending on its particular implementation.

## 1. Introduction

Linear machine generates translations directly without rotation-to-transmission conversion mechanisms, and thus achieves compact structure, high efficiency and good dynamic performance. It has wide applications in aerospace industries [1,2], transportation [3,4], high-precision manufacture [5,6], energy harvesting [7,8], robotics [9,10], and medical operations. Linear machine can be used as either generator or actuator to accomplish different works. 

Magnetic flux density is extremely important for the design of linear machines. High flux density helps to increase force output or signal strength depending on particular tasks. The conventional method is to increase the magnet size and thus the flux density. However, the large size or mass of the system is apparently not preferred for most applications, especially in aerospace technology. Alternatively, various magnet patterns have been proposed by researchers to enhance the magnetic flux density inside the linear machines without changing the system size significantly. Buren et al. [11] presents one linear electromagnetic generator with three axially magnetized PM poles. The relative motion between stator and translator leads to a varying magnetic flux through the armature windings, and the output power proportional to the rate of flux change is induced. Several axially magnetized disc-shaped magnets separated by soft-magnetic spacers are mounted on the translator. The magnetization directions of neighboring magnets are opposite, and the spacers act as flux concentrators and form the magnet poles. The design may lead to high flux leakage, and decrease the force output. An improved version of axial magnetization is proposed by Kim et al. [12] in a tubular linear brushless machine. Cylindrical permanent magnets are placed in an NS-NS-SN-SN fashion with spacers between pairs. The magnets are fixed within a freely sliding brass tube on the mover. Coils are configured in three phases to interact with the magnet and cause translation of the mover. This structure and magnetization pattern help to increase flux density near the like pole regions. However, the aluminum tubes used to separate magnets decrease the volume efficiency of the mover. Huang et al. [13] employed convex pole instead of rectangular pole in the axial magnetization pattern to reduce the magnetic saturation and flux leakage of tubular machines. High thrust and less permanent magnet dosage were achieved in the research. However, the magnetization and assembly are relatively challenging. Wang et al. [14] analyzed a tubular linear machine with surface-mounted radially magnetized magnets and came to the conclusion that this pattern can reduce the magnet material and the cost. Special customized fixture may be required for the magnetization. Nirei et al. [15] developed a moving-coil linear machine with typical radial magnetization. There were 16 pieces of permanent magnets mounted along the inner surface of the outer yoke. Due to the same polarization, the multiple magnets are essentially equivalent to one magnet ring with radial magnetization. However, the single-ring pattern unavoidably causes high flux leakage and reduces the force output. Baloch et al. [16] designed a tubular vernier machine adopting dual stator configurations. Multiple PM poles are mounted on the mover along the axial direction. The poles are alternatively magnetized radially, which helps to achieve high force output and large working range compared with the design in [15]. Wang et al. [17] presented a novel linear electromagnetic machine based on the concept of magnetic screw-nut. The radially magnetized permanent magnets are helically disposed on the nut and the screw to produce force and torque simultaneously. However, the two motions are coupled, and cannot be controlled independently. The Halbach array was firstly proposed a few decades ago [18], and implemented into design of linear machines recently [19]. It can enhance the flux density on the one side of PM, and reduce the flux leakage on the other side in a certain degree. Yan et al. [20] proposed a high dynamic performance linear machine with improved Halbach array. A combination of two axial magnets and one radial magnet is utilized in the design of magnet topology. Compared with commonly used quasi-Halbach array, it enhances the self-shielding effect without the utilization of back irons. Therefore, the mover mass is reduced significantly, and thus the dynamic response is increased greatly. Izzeldin [21] analyzed the magnet patterns of three moving-magnet linear machines, including rectangular, trapezoidal and T-shape magnet arrays with quasi-Halbach magnetization. It is shown that the T-shape and trapezoidal magnet arrays achieve better flux linkage than the rectangular one. However, the fabrication and magnetization of magnets are relatively challenging. Yan et al. [22,23] extended conventional magnet array into three-dimensional topology, and proposed the novel dual Halbach array. It helps to improve the axial force output and depress the radial vibration. Magnet patterns have also been employed to improve the performance of flux-switching machines. For example, Zhang et al. [24] proposed a yokeless linear machine topology with double magnets per mover module to enhance thrust density but efficiency of the new machine is marginally higher than that of the conventional yokeless machine. The comparison of magnet patterns is summarized in Table 1.

The objective of this study is to propose one novel linear permanent magnet machine with three-layer hybrid magnets and two-layer windings. The employment of the proposed structure offers the following advantages. The combination of conventional Halbach array and alternatively magnetized radial poles helps to enhance the magnetic flux density in the radial direction, and thus increase the force output of the linear machine. In addition, the utilization of multiple movers in the linear machine increases the redundancy and reliability of the system, which is extremely important for the aerospace technology. Furthermore, the winding mass is reduced, and thus the dynamic response could be improved significantly for moving coil designs. The design concept is presented, Laplace’s and Poisson’s equations are obtained from source free property of magnetic field and Maxwell equations. The general solution of magnetic vector potential is represented with Bessel functions. The flux distribution inside the machine is then formulated analytically by utilizing harmonic expansion of magnetization vectors and boundary conditions. Numerical computation is conducted to validate the analytical model and on the proposed design.

## 2. Concept Design

**Figure 1:**
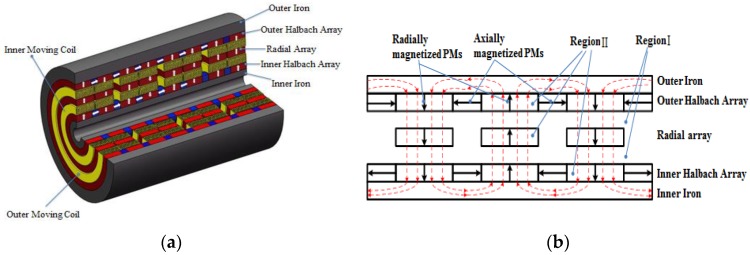
Tubular linear machine with hybrid magnet arrays: (**a**) Schematic machine structure; (**b**) Magnetization and flux.

The schematic structure of the proposed tubular linear machine is illustrated in Figure 1a. It consists of two layers of windings and three layers of magnet poles. The windings are mounted on the mover, and the magnet poles are on the stator. The relative motion between the stator and mover generates voltages on the windings proportional to the motion speed. It is worth pointing out that the two layers of windings could be connected together to enhance the voltage signal, or separated to detect motions of individual rigid bodies. The redundancy property improves the reliability of the linear machines. The multiple-winding structure also benefits the heat dissipation. The same structure can be utilized for design of actuators in aircrafts. Magnetization pattern of the hybrid magnet arrays is illustrated in Figure 1b. One layer of radially magnetized PM poles are placed in between the two layers of Halbach arrays. All radially magnetized PM poles in the three layers have the same polarization while the axial PMs of two Halbach layers are magnetized with opposite directions. The proposed hybrid arrangement can increase the flux density in the radial direction greatly. Therefore, the axial force generation is improved due to the cross product of flux density and current input.

## 3. Governing Equations

In this section, the flux field in the proposed linear machine is formulated analytically. The obtained mathematical model could be employed for subsequent design optimization, modeling of thrust or output current, and high precision motion control.

### 3.1. Assumptions

(a) The machine has a periodic magnetic structure along axial direction *z*.
(b) The axial length is infinite, and thus the end effects can be ignored.
(c) The distribution of magnetic field is axially symmetric.
(d) The magnetic permeability of back iron is infinite.

### 3.2. Characterization of Materials and Governing Equations

**Figure 2:**
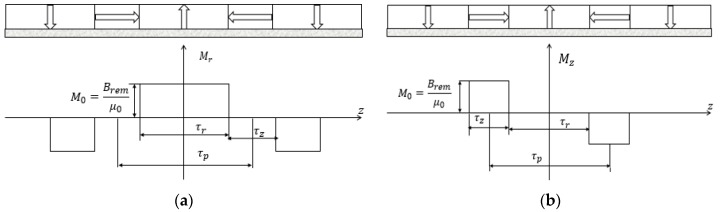
Components of magnetization vector of Halbach layers: (**a**) Radial component; (**b**) Axial component.

To formulate the magnetic field mathematically, the space in linear machine under study is divided into two regions based on their magnetic characteristics. The air space is denoted as Region I. The PM pole filled with rare-earth magnetic material is denoted as Region II (Figure 2). According to different material properties, it is easy to obtain equations relating magnetic field intensity H(A/m) to flux density **B**(T) for these two regions

(1) $$B=\mu_{0}H,\;B=\mu_{0}\mu_{r}H+\mu_{0}M,$$

where $\mu_{0}$ is the permeability of vacuum with a value of $4\pi \times 10^{-7}\left(N/A^{2}\right)$, dimensionless quantity $\mu_{r}$ is the relative permeability of permanent magnets, and M = $B_{\mathrm{rem}}/\mu_{0}$ (A/m) is the residual magnetization vector. **B** is equal to the curl of magnetic vector potential, i.e.,

(2) ∇×A=B.

The Coulomb gauge is used as a constraint to uniquely determine the divergence of a vector. As a result, we have Laplace’s Equation, or governing equation for region I

(3) $$\nabla^{2}A=0$$

and Poisson’s Equation, for region II

(4) $$\nabla^{2}A=-\mu_{0}\nabla \times M.$$

## 4. Formulation of Magnetic Field 

### 4.1. General Solution to Magnetic Potential in Region I

Based on the symmetric distribution of flux density in tubular linear machines, the Laplace’s equation, i.e., Equation (3) in cylindrical coordinates can be expanded and simplified as

(5) $$\frac{\partial }{\partial r}\left(\frac{1}{r}\frac{\partial \left(rA_{\theta }\right)}{\partial r}\right)+\frac{\partial^{2}A_{\theta }}{\partial z^{2}}=0.$$

Since $A_{\theta }$ is only a function of independent variables, *r* and *z*, it can be represented with separation principle of variables as

(6) $$A_{\theta }=R\left(r\right)Z\left(z\right).$$

Substituting Equation (6) into (5) gives

(7) $$\frac{1}{R\left(r\right)}\frac{\partial^{2}R\left(r\right)}{\partial r^{2}}+\frac{1}{R\left(r\right)r}\frac{\partial R\left(r\right)}{\partial r}-\frac{1}{r^{2}}+\frac{1}{Z\left(z\right)}\frac{\partial^{2}Z\left(z\right)}{\partial z^{2}}=0.$$

$A_{\theta }\;$is determined uniquely based on the structure of tubular linear machines. The magnetic field in tubular linear machines is periodically and symmetrically distributed along *z* axial, thus, the solution to magnetic potential in Region I is

(8) $$A_{\theta }=\sum_{n=1}^{\infty }\left[a_{n}I_{1}\left(m_{n}r\right)+b_{n}K_{1}\left(m_{n}r\right)\right]\sin\left(m_{n}z\right),$$

where $I_{1}$ and $K_{1}$ are the modified Bessel functions of the first and second kind [25], $m_{n}=\frac{\left(2n-1\right)\pi }{\tau_{p}}$, *n* is a positive integer.

### 4.2. General Solution to Magnetic Potential in Region II

For the specific structure of tubular linear machine under study in this paper, Poisson’s Equation in cylindrical coordinates can be expanded as

(9) $$\frac{\partial }{\partial r}\left(\frac{1}{r}\frac{\partial \left(rA_{\theta }\right)}{\partial r}\right)+\frac{\partial^{2}A_{\theta }}{\partial z^{2}}=-\mu_{0}\left(\frac{\partial M_{r}}{\partial z}-\frac{\partial M_{z}}{\partial r}\right),$$

where $M_{r}$ and $M_{z}$ are the radial and axial components of magnetization vector M, respectively. The homogeneous solution to Poisson’s Equation is exactly the same as the general solution to Laplace’s equation. Therefore, deriving the particular solution of Equation (9) will determine the general solution of Poisson’s equation. To formulate the particular solution, we need to substitute the right side of Equation (9) with its harmonic expansion. As shown in Figure 1, the two layers of Halbach arrays in the proposed novel linear machine are composed of axial and radial magnets whereas the middle layer only has radially magnetized magnets. 

As illustrated in Figure 2a, $M_{r}$ is a non-continuous periodic even function with a period of $2\tau_{p}$. Its harmonic expansion is

(10) $$M_{r}=\sum_{n=1}^{\infty }\frac{4B_{rem}}{\left(2n-1\right)\pi \mu_{0}}\sin\left(\frac{\left(2n-1\right)\pi \tau_{r}}{2\tau_{p}}\right)\cos\left(m_{n}z\right),$$

where $\tau_{p}$ is the pole pitch, $\tau_{r}$ is the width of radial magnets, and $B_{rem}$ is the remanence. As shown in Figure 2b, $M_{z}$ is a non-continuous periodic odd function with a period of $2\tau_{p}$. Its harmonic expansion is

(11) $$M_{z}=-\sum_{n=1}^{\infty }\frac{4B_{rem}}{\left(2n-1\right)\pi \mu_{0}}\cos\left(\frac{\left(2n-1\right)\pi \tau_{r}}{2\tau_{p}}\right)\sin\left(m_{n}z\right),$$

where $\tau_{z}$ is the width of axial magnets. 

**Figure 3:**
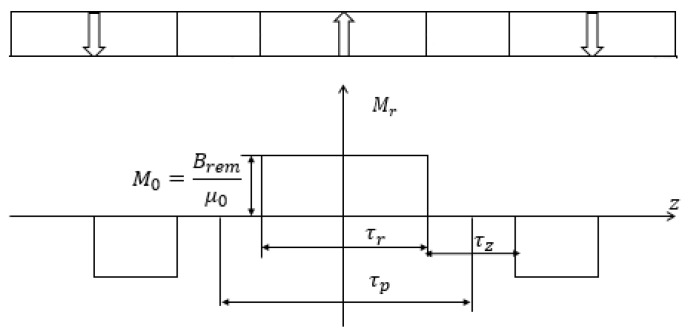
Component of magnetization vector of middle layer.

The magnetization pattern of the middle PM layer is exactly the same as the radial magnets of Halbach layers (Figure 3). Therefore, the radial component of magnetization vector can be expressed with Equation (10). Substituting Equations (10) and (11) into (9) yields the following form of Poisson’s equation for Halbach arrays and radial PM array, i.e.,

(12) $$\frac{\partial }{\partial r}\left(\frac{1}{r}\frac{\partial \left(rA_{\theta }\right)}{\partial r}\right)+\frac{\partial^{2}A_{\theta }}{\partial z^{2}}=\sum_{n=1}^{\infty }\frac{4B_{rem}}{\tau_{p}}\sin\left(\frac{\left(2n-1\right)\pi \tau_{r}}{2\tau_{p}}\right)\sin\left(m_{n}z\right).$$

Thus, the general solution to Poisson’s equation is

(13) $$A_{\theta }=\sum_{n=1}^{\infty }\left\{\left[a_{n}I_{1}\left(m_{n}r\right)+b_{n}K_{1}\left(m_{n}r\right)\right]\sin\left(m_{n}z\right)+s\left(r,z\right)\right\}.$$

The homogeneous part of Equation (13) is the same as Equation (8) and the particular part s(r,z) can be derived by using separation of variables as

(14) $$s\left(r,z\right)=L_{1}\left(m_{n}r\right)\frac{\pi P_{n}}{2m_{n}{}^{2}}\sin\left(m_{n}z\right),$$

where $L_{1}\left(m_{n}r\right)$ are the modified Struve functions [26]. Therefore, the general solution to Poisson’s equation is

(15) $$A_{\theta }=\sum_{n=1}^{\infty }\left\{\left[a_{n}I_{1}\left(m_{n}r\right)+b_{n}K_{1}\left(m_{n}r\right)\right]\sin\left(m_{n}z\right)+L_{1}\left(m_{n}r\right)\frac{\pi P_{n}}{2m_{n}{}^{2}}\sin\left(m_{n}z\right)\right\}.$$

### 4.3. Analytical Model of Flux Density

From Equations (1), (8) and (15), the general solution of flux density is obtained

(16) $$B_{r1}^{i}=-\frac{\partial A_{\theta }}{\partial z}=-\sum_{n=1}^{\infty }m_{n}\left[a_{1n}^{i}I_{1}\left(m_{n}r\right)+b_{1n}^{i}K_{1}\left(m_{n}r\right)\right]\cos\left(m_{n}z\right)$$

(17) $$B_{z1}^{i}=\frac{A_{\theta }}{r}+\frac{\partial A_{\theta }}{\partial r}=\overset{\infty }{\underset{n=1}{\sum }}m_{n}\left[a_{1n}^{i}I_{0}\left(m_{n}r\right)-b_{1n}^{i}K_{0}\left(m_{n}r\right)\right]\sin\left(m_{n}z\right)\;i=1,2;$$

(18) $$B_{r2}^{j}=-\overset{\infty }{\underset{n=1}{\sum }}m_{n}\left\{\left[a_{2n}^{j}I_{1}\left(m_{n}r\right)+b_{2n}^{j}K_{1}\left(m_{n}r\right)+L_{1}\left(m_{n}r\right)\frac{\pi P_{n}}{2m_{n}{}^{2}}\right]\cos\left(m_{n}z\right))\right\}$$

(19) $$B_{z2}^{j}=\overset{\infty }{\underset{n=1}{\sum }}m_{n}\left\{\left[a_{2n}^{j}I_{0}\left(m_{n}r\right)-b_{2n}^{j}K_{0}\left(m_{n}r\right)+L_{0}\left(m_{n}r\right)\frac{\pi P_{n}}{2m_{n}{}^{2}}\right]\sin\left(m_{n}z\right))\right\}\;j=1,2,3.$$

$B_{r1}^{i}$ and $B_{z1}^{i}$ represent the radial and axial magnetic field in the air region, respectively, while $B_{r2}^{j}$ and $B_{z2}^{j}$ are in magnet regions. The upper script, *i* = 1, 2, represents two sections of air region, while *j* = 1, 2, 3 represents internal, middle and external PMs respectively.

### 4.4. Boundary Conditions

**Figure 4:**
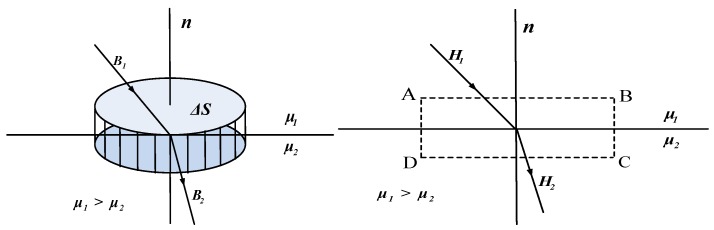
Boundary conditions of flux density and field intensity.

There are 10 unknown coefficients in the analytical model of flux density, i.e., $a_{1n}^{1},\;a_{1n}^{2}$, $b_{1n}^{1}$, $b_{1n}^{2}$, $a_{2n}^{1}$, $b_{2n}^{1}$, $a_{2n}^{2}$, $b_{2n}^{2}$, $a_{2n}^{3}$ and$\;b_{2n}^{3}$. As shown in Figure 4, based on the continuity of flux density and Ampere circuital theorem, the perpendicular component of flux density is continuous in two adjacent media and the tangential component of field intensity is continuous at the boundary of two media when the surface current is zero [27,28]. Ten boundary conditions are employed to determine all the unknowns, i.e.,

(20) $${H_{z2}^{1}|}_{r=R_{1}}=0;\;{H_{z2}^{3}|}_{r=R_{6}}=0;\;{H_{z2}^{1}|}_{r=R_{2}}={H_{z1}^{1}|}_{r=R_{2}};\;{B_{r2}^{1}|}_{r=R_{2}}={B_{r1}^{1}|}_{r=R_{2}};\;{H_{z2}^{2}|}_{r=R_{3}}={H_{z1}^{1}|}_{r=R_{3}};\;{B_{r2}^{2}|}_{r=R_{3}}={B_{r1}^{1}|}_{r=R_{3}};\;{H_{z2}^{2}|}_{r=R_{4}}={H_{z1}^{2}|}_{r=R_{4}};\;{B_{r2}^{2}|}_{r=R_{4}}={B_{r1}^{2}|}_{r=R_{4}};\;{H_{z2}^{3}|}_{r=R_{5}}={H_{z1}^{2}|}_{r=R_{5}};\;{B_{r2}^{3}|}_{r=R_{5}}={B_{r1}^{2}|}_{r=R_{5}},$$

where

(21) $$H_{z1}^{i}=\frac{B_{z1}^{i}}{\mu_{0}}=\frac{\sum_{n=1}^{\infty }m_{n}\left[a_{1n}^{i}I_{0}\left(m_{n}r\right)-b_{1n}^{i}K_{0}\left(m_{n}r\right)\right]\sin\left(m_{n}z\right)}{\mu_{0}}\;H_{z2}^{j}=\frac{\sum_{n=1}^{\infty }m_{n}\left\{\left[a_{2n}^{j}I_{0}\left(m_{n}r\right)-b_{2n}^{j}K_{0}\left(m_{n}r\right)+L_{0}\left(m_{n}r\right)\frac{\pi P_{n}}{2m_{n}{}^{2}}\right]\sin\left(m_{n}z\right))\right\}}{\mu_{0}\mu_{r}}-\frac{M_{z2}^{j}}{\mu_{r}}\;M_{z2}^{1}=-M_{z2}^{3}=-\overset{\infty }{\underset{n=1}{\sum }}\frac{4B_{rem}}{\left(2n-1\right)\pi \mu_{0}}\cos\left(\frac{\left(2n-1\right)\pi \tau_{r}}{2\tau_{p}}\right)\sin\left(m_{n}z\right)\;M_{z2}^{2}=0$$

**Figure 5:**
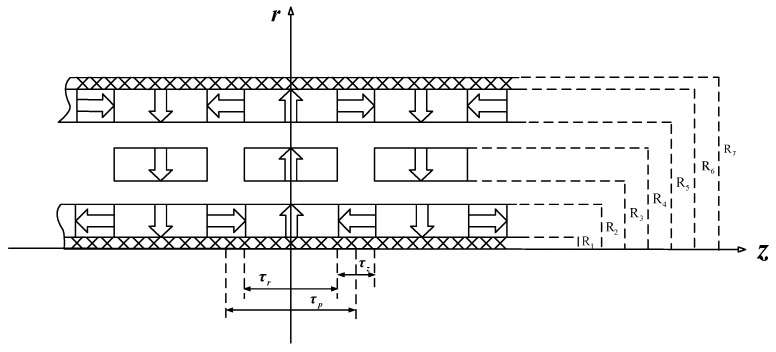
Major structural parameters of the proposed linear machine.

The major structural parameters in the boundary conditions are presented in Figure 5. 

Substituting Equations (16)–(18), (19) and (21) into Equation (20) will derive ten expanded equations describing ten boundary conditions respectively. They are shown as follows:

(22a) $$a_{2n}^{1}I_{0}\left(m_{n}R_{1}\right)-b_{2n}^{1}K_{0}\left(m_{n}R_{1}\right)=-L_{0}\left(m_{n}R_{1}\right)\frac{\pi P_{n}}{2m_{n}{}^{2}}-\frac{4B_{rem}}{\left(2n-1\right)\pi m_{n}}\cos\left(\frac{\left(2n-1\right)\pi \tau_{r}}{2\tau_{p}}\right),$$

(22b) $$a_{2n}^{3}I_{0}\left(m_{n}R_{6}\right)-b_{2n}^{3}K_{0}\left(m_{n}R_{6}\right)=-L_{0}\left(m_{n}R_{6}\right)\frac{\pi P_{n}}{2m_{n}{}^{2}}+\frac{4B_{rem}}{\left(2n-1\right)\pi m_{n}}\cos\left(\frac{\left(2n-1\right)\pi \tau_{r}}{2\tau_{p}}\right),$$

(22c) $$\mu_{r}a_{1n}^{1}I_{0}\left(m_{n}R_{2}\right)-\mu_{r}b_{1n}^{1}K_{0}\left(m_{n}R_{2}\right)-a_{2n}^{1}I_{0}\left(m_{n}R_{2}\right)+b_{2n}^{1}K_{0}\left(m_{n}R_{2}\right)\;=L_{0}\left(m_{n}R_{2}\right)\frac{\pi P_{n}}{2m_{n}{}^{2}}+\frac{4B_{rem}}{\left(2n-1\right)\pi m_{n}}\cos\left(\frac{\left(2n-1\right)\pi \tau_{r}}{2\tau_{p}}\right),$$

(22d) $$a_{2n}^{1}I_{1}\left(m_{n}R_{2}\right)+b_{2n}^{1}K_{1}\left(m_{n}R_{2}\right)-a_{1n}^{1}I_{1}\left(m_{n}R_{2}\right)-b_{1n}^{1}K_{1}\left(m_{n}R_{2}\right)=-L_{1}\left(m_{n}R_{2}\right)\frac{\pi P_{n}}{2m_{n}{}^{2}},$$

(22e) $$\mu_{r}a_{1n}^{1}I_{0}\left(m_{n}R_{3}\right)-\mu_{r}b_{1n}^{1}K_{0}\left(m_{n}R_{3}\right)-a_{2n}^{2}I_{0}\left(m_{n}R_{3}\right)+b_{2n}^{2}K_{0}\left(m_{n}R_{3}\right)=L_{0}\left(m_{n}R_{3}\right)\frac{\pi P_{n}}{2m_{n}{}^{2}},$$

(22f) $$a_{2n}^{2}I_{1}\left(m_{n}R_{3}\right)+b_{2n}^{2}K_{1}\left(m_{n}R_{3}\right)-a_{1n}^{1}I_{1}\left(m_{n}R_{3}\right)-b_{1n}^{1}K_{1}\left(m_{n}R_{3}\right)=-L_{1}\left(m_{n}R_{3}\right)\frac{\pi P_{n}}{2m_{n}{}^{2}},$$

(22g) $$\mu_{r}a_{1n}^{2}I_{0}\left(m_{n}R_{4}\right)-\mu_{r}b_{1n}^{2}K_{0}\left(m_{n}R_{4}\right)-a_{2n}^{2}I_{0}\left(m_{n}R_{4}\right)+b_{2n}^{2}K_{0}\left(m_{n}R_{4}\right)=L_{0}\left(m_{n}R_{4}\right)\frac{\pi P_{n}}{2m_{n}{}^{2}},$$

(22h) $$a_{2n}^{2}I_{1}\left(m_{n}R_{4}\right)+b_{2n}^{2}K_{1}\left(m_{n}R_{4}\right)-a_{1n}^{2}I_{1}\left(m_{n}R_{4}\right)-b_{1n}^{2}K_{1}\left(m_{n}R_{4}\right)=-L_{1}\left(m_{n}R_{4}\right)\frac{\pi P_{n}}{2m_{n}{}^{2}},$$

(22i) $$\mu_{r}a_{1n}^{2}I_{0}\left(m_{n}R_{5}\right)-\mu_{r}b_{1n}^{2}K_{0}\left(m_{n}R_{5}\right)-a_{2n}^{3}I_{0}\left(m_{n}R_{5}\right)+b_{2n}^{3}K_{0}\left(m_{n}R_{5}\right)=\;L_{0}\left(m_{n}R_{5}\right)\frac{\pi P_{n}}{2m_{n}{}^{2}}-\frac{4B_{rem}}{\left(2n-1\right)\pi m_{n}}\cos\left(\frac{\left(2n-1\right)\pi \tau_{r}}{2\tau_{p}}\right),$$

(22j) $$a_{2n}^{3}I_{1}\left(m_{n}R_{5}\right)+b_{2n}^{3}K_{1}\left(m_{n}R_{5}\right)-a_{1n}^{2}I_{1}\left(m_{n}R_{5}\right)-b_{1n}^{2}K_{1}\left(m_{n}R_{5}\right)=-L_{1}\left(m_{n}R_{5}\right)\frac{\pi P_{n}}{2m_{n}{}^{2}},$$

The matrix form of Equations (22a) until (22j) is

(23) $$AX=F\;\left[\begin{array}{cccccccccc} 0 & 0 & 0 & 0 & A_{15} & A_{16} & 0 & 0 & 0 & 0\\ 0 & 0 & 0 & 0 & 0 & 0 & 0 & 0 & A_{29} & A_{210}\\ A_{31} & A_{32} & 0 & 0 & A_{35} & A_{36} & 0 & 0 & 0 & 0\\ A_{41} & A_{42} & 0 & 0 & A_{45} & A_{46} & 0 & 0 & 0 & 0\\ A_{51} & A_{52} & 0 & 0 & 0 & 0 & A_{57} & A_{58} & 0 & 0\\ A_{61} & A_{62} & 0 & 0 & 0 & 0 & A_{67} & A_{68} & 0 & 0\\ 0 & 0 & A_{73} & A_{74} & 0 & 0 & A_{77} & A_{78} & 0 & 0\\ 0 & 0 & A_{83} & A_{84} & 0 & 0 & A_{87} & A_{88} & 0 & 0\\ 0 & 0 & A_{93} & A_{94} & 0 & 0 & 0 & 0 & A_{99} & A_{910}\\ 0 & 0 & A_{103} & A_{104} & 0 & 0 & 0 & 0 & A_{109} & A_{1010} \end{array}][\begin{array}{c} X_{1}\\ X_{2}\\ X_{3}\\ X_{4}\\ X_{5}\\ X_{6}\\ X_{7}\\ X_{8}\\ X_{9}\\ X_{10} \end{array}]=[\begin{array}{c} F_{1}\\ F_{2}\\ F_{3}\\ F_{4}\\ F_{5}\\ F_{6}\\ F_{7}\\ F_{8}\\ F_{9}\\ F_{10} \end{array}\right],$$

where

$$A_{15}=I_{0}\left(m_{n}R_{1}\right);\;A_{16}=-K_{0}\left(m_{n}R_{1}\right);\;F_{1}=-L_{0}\left(m_{n}R_{1}\right)\frac{\pi P_{n}}{2m_{n}{}^{2}}-\frac{4B_{rem}}{\left(2n-1\right)\pi m_{n}}\cos\left(\frac{\left(2n-1\right)\pi \tau_{r}}{2\tau_{p}}\right);\;\;A_{29}=I_{0}\left(m_{n}R_{6}\right);\;A_{210}=-K_{0}\left(m_{n}R_{6}\right);\;F_{2}=-L_{0}\left(m_{n}R_{6}\right)\frac{\pi P_{n}}{2m_{n}{}^{2}}+\frac{4B_{rem}}{\left(2n-1\right)\pi m_{n}}\cos\left(\frac{\left(2n-1\right)\pi \tau_{r}}{2\tau_{p}}\right);\;\;A_{31}=\mu_{r}I_{0}\left(m_{n}R_{2}\right);\;A_{32}=-\mu_{r}K_{0}\left(m_{n}R_{2}\right);\;A_{35}=-I_{0}\left(m_{n}R_{2}\right);\;A_{36}=K_{0}\left(m_{n}R_{2}\right);\;\;F_{3}=L_{0}\left(m_{n}R_{2}\right)\frac{\pi P_{n}}{2m_{n}{}^{2}}+\frac{4B_{rem}}{\left(2n-1\right)\pi m_{n}}\cos\left(\frac{\left(2n-1\right)\pi \tau_{r}}{2\tau_{p}}\right);\;A_{45}=I_{1}\left(m_{n}R_{2}\right);\;A_{46}=K_{1}\left(m_{n}R_{2}\right);\;\;A_{41}=-I_{1}\left(m_{n}R_{2}\right);\;A_{42}=-K_{1}\left(m_{n}R_{2}\right);\;F_{4}=-L_{1}\left(m_{n}R_{2}\right)\frac{\pi P_{n}}{2m_{n}{}^{2}};\;A_{51}=\mu_{r}I_{0}\left(m_{n}R_{3}\right);\;\;A_{52}=-\mu_{r}K_{0}\left(m_{n}R_{3}\right);\;A_{57}=-I_{0}\left(m_{n}R_{3}\right);\;A_{58}=K_{0}\left(m_{n}R_{3}\right);\;F_{5}=L_{0}\left(m_{n}R_{3}\right)\frac{\pi P_{n}}{2m_{n}{}^{2}};\;\;A_{67}=I_{1}\left(m_{n}R_{3}\right);\;A_{68}=K_{1}\left(m_{n}R_{3}\right);\;A_{61}=-I_{1}\left(m_{n}R_{3}\right);\;A_{62}=-K_{1}\left(m_{n}R_{3}\right);\;\;F_{6}=-L_{1}\left(m_{n}R_{3}\right)\frac{\pi P_{n}}{2m_{n}{}^{2}};\;A_{73}=\mu_{r}I_{0}\left(m_{n}R_{4}\right);\;A_{74}=-\mu_{r}K_{0}\left(m_{n}R_{4}\right);\;A_{77}=-I_{0}\left(m_{n}R_{4}\right);\;\;A_{78}=K_{0}\left(m_{n}R_{4}\right);\;F_{7}=L_{0}\left(m_{n}R_{4}\right)\frac{\pi P_{n}}{2m_{n}{}^{2}};\;A_{87}=I_{1}\left(m_{n}R_{4}\right);\;A_{88}=K_{1}\left(m_{n}R_{4}\right);\;A_{83}=-I_{1}\left(m_{n}R_{4}\right);\;\;A_{84}=-K_{1}\left(m_{n}R_{4}\right);\;F_{8}=-L_{1}\left(m_{n}R_{4}\right)\frac{\pi P_{n}}{2m_{n}{}^{2}};\;A_{93}=\mu_{r}I_{0}\left(m_{n}R_{5}\right);\;A_{94}=-\mu_{r}K_{0}\left(m_{n}R_{5}\right);\;\;A_{99}=-I_{0}\left(m_{n}R_{5}\right);\;A_{910}=K_{0}\left(m_{n}R_{5}\right);\;F_{9}=L_{0}\left(m_{n}R_{5}\right)\frac{\pi P_{n}}{2m_{n}{}^{2}}-\frac{4B_{rem}}{\left(2n-1\right)\pi m_{n}}\cos\left(\frac{\left(2n-1\right)\pi \tau_{r}}{2\tau_{p}}\right);\;\;A_{109}=I_{1}\left(m_{n}R_{5}\right);\;A_{1010}=K_{1}\left(m_{n}R_{5}\right);\;A_{103}=-I_{1}\left(m_{n}R_{5}\right);\;A_{104}=-K_{1}\left(m_{n}R_{5}\right);\;\;F_{10}=-L_{1}\left(m_{n}R_{5}\right)\frac{\pi P_{n}}{2m_{n}{}^{2}}.$$

## 5. Numerical Simulation and Analysis

### 5.1. Numerical Model

The numerical approach is an effective way to analyze the magnetic flux field distribution of electromagnetic actuators. The values of major parameters for numerical computation are listed in Table 2. These parameter values are obtained by maximizing the force output. With the values in Table 2, the maximum force output is 112 N. The details of design optimization based on force model and motion control implementation with force feedback will be covered in another paper.

**Figure 6:**
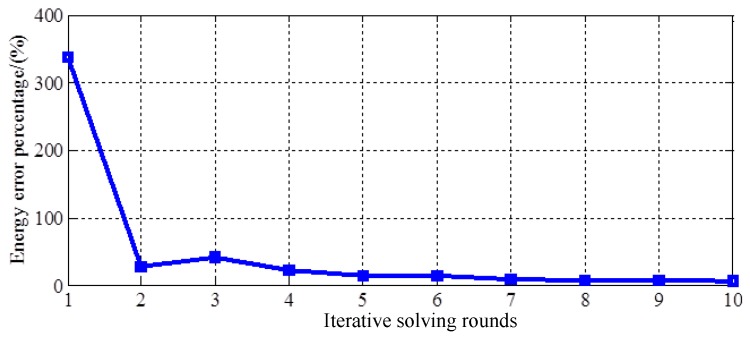
Relation between iterative solving rounds and energy error percentage.

The structure of the proposed linear machine is axially symmetric, so Magnetostatic 2D solver is conducted on Maxwell to model and analyze the flux field. In this study, the whole model is divided into 179,958 mesh elements to derive accurate solution, with the RMS edge length ranging from 0.00012 mm to 0.00051 mm for different parts of solving regions. The relationship between the number of iterative solving rounds and the energy error percentage is shown in Figure 6. The energy error percentage is used to represent the accuracy of numerical solution in Maxwell. It can be found that after 10 rounds of iterative solving, the energy error is below 5%, and it is acceptable for analysis and validation of electromagnetic machines.

**Figure 7:**
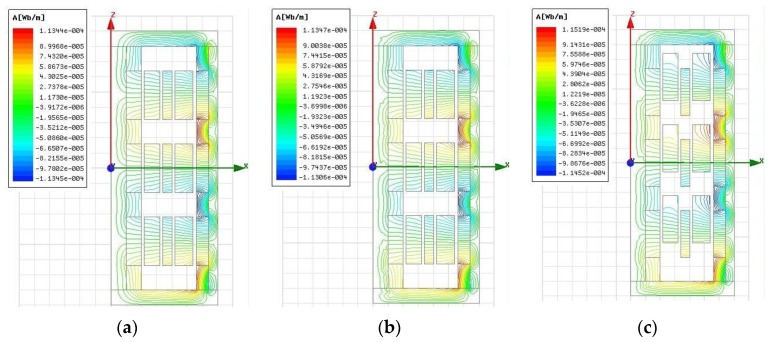
Magnetic flux distribution: (**a**) Initial position without power supply; (**b**) Initial position with power supply; (**c**) Maximum working position with power supply.

Flux distribution corresponding to the initial and maximum working positions of coils without and with load is shown in Figure 7a–c respectively. It can be found that the magnetic flux is generated by the permanent magnet, goes across the air gap filled with windings, and returns through the back irons, forming a close loop. As indicated in Figure 7b,c, flux distribution varies for different coil positions, but is not affected significantly by power supply of coils.

**Figure 8:**
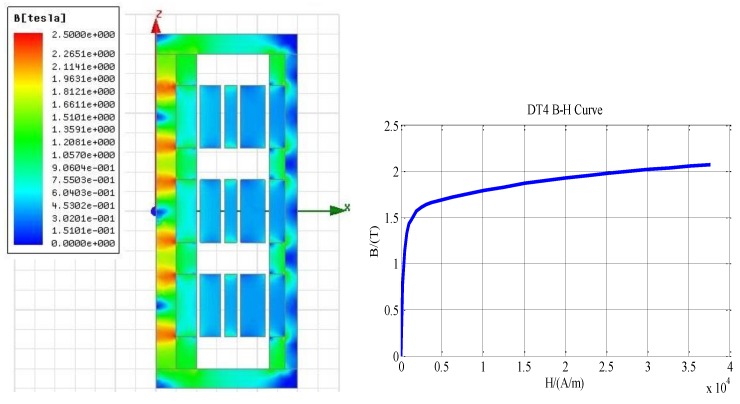
Flux density in the linear machine and BH curve of back iron.

Figure 8 illustrated the BH curve to analyze magnetic saturation in the machine. It is found that there is no significant saturation in the system. Only slight saturation exists at the inner back iron due to the relatively small volume. Further design optimization might be conducted to reduce the saturation if necessary.

**Figure 9:**
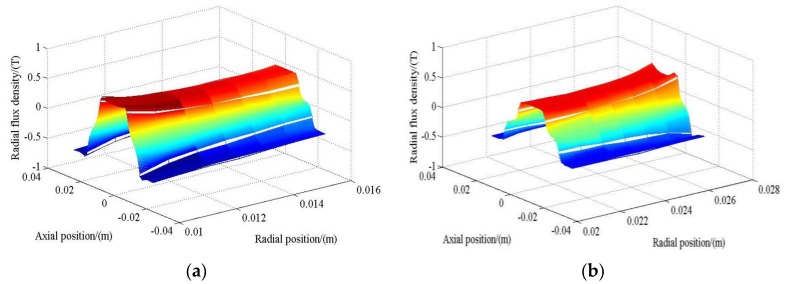
Magnetic flux density with respect to axial and radial directions: (**a**) Inner winding region; (**b**) Outer winding region.

To precisely observe the variation of magnetic flux field, Figure 9a,b show the flux distribution with respect to axial and radial positions in outer and inner air gaps, respectively. Only the radial flux component is presented, because only this component can produce voltage signal or axial thrust in this machine. Figure 9a shows that the radial flux density does not change significantly in the radial direction. However, it is slightly larger at positions close to the inner magnet layer. Similarly, Figure 9b indicates that the flux density close to the outer layer of Halbach array is relatively larger. In the axial direction, the flux density for both air gaps varies in trigonometric form, which is consistent with the magnetization pattern of PM poles in this direction.

### 5.2. Validation of Analytical Models

**Figure 10:**
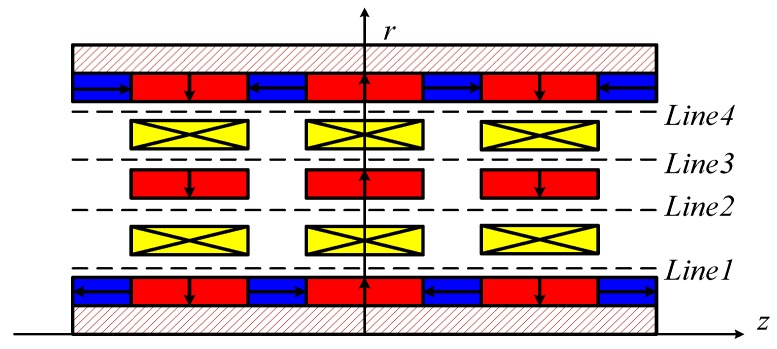
Positions for comparison of numerical result and analytical models.

The analytical model is a powerful tool for design optimization and control implementation of electromagnetic actuators. In contrast, the numerical approach is an efficient and reliable way to validate analytical results. To compare the numerical result and analytical model precisely, four positions indicated by lines in the air region are utilized for simulation, as shown in Figure 10. 

**Figure 11:**
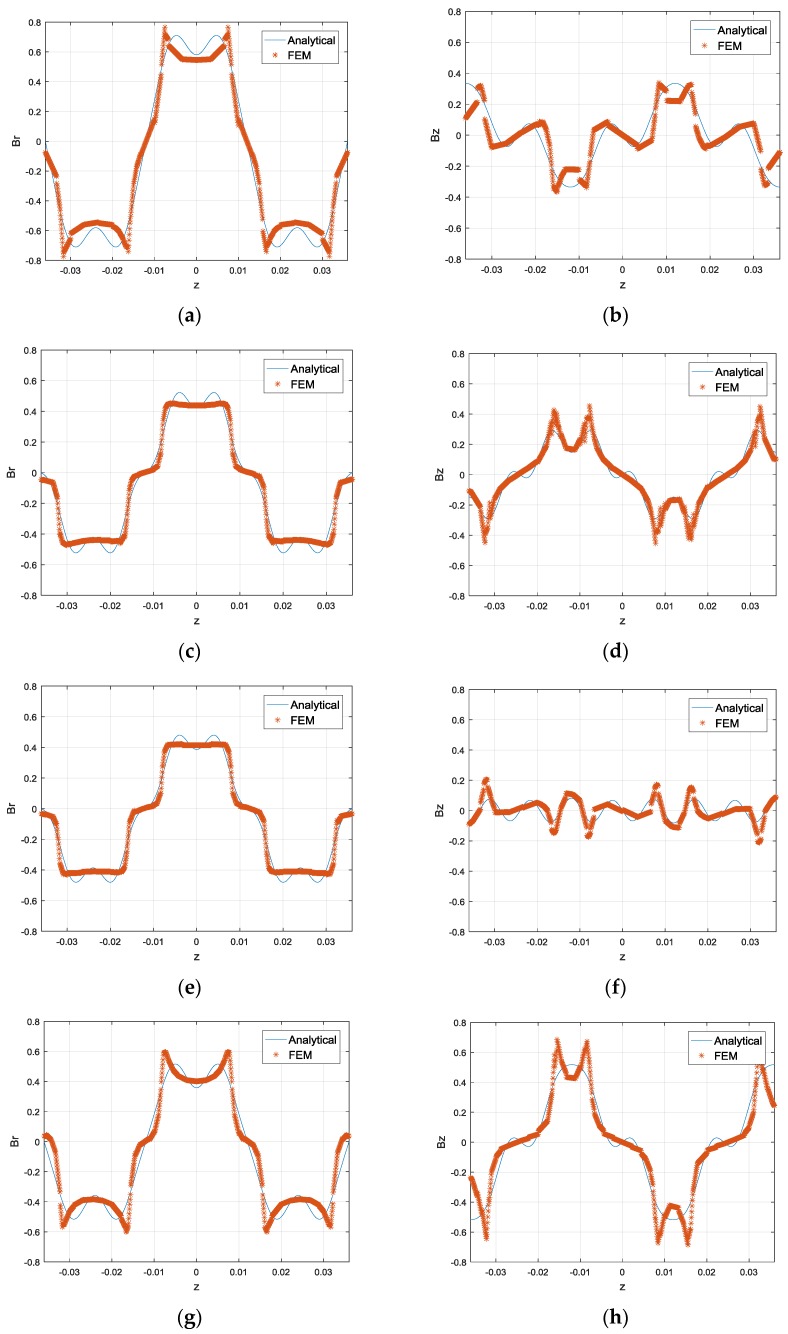
Comparison of analytical model and numerical result: (**a**) Radial flux density on line 1; (**b**) Axial flux density on line 1. (**c**) Radial flux density on line 2; (**d**) Axial flux density on line 2. (**e**) Radial flux density on line 3; (**f**) Axial flux density on line 3. (**g**) Radial flux density on line 4; (**h**) Axial flux density on line 4.

The comparison result of the analytical model and the numerical computation is presented in Figure 11. Figure 11a,b represent the variation of flux component in radial and axial directions, respectively, for Line 1. Similarly, the flux variation for Line 2–4 is presented in Figure 11c–h. It is found that the analytical model fits with the numerical result well. It could be employed for the study on modeling of force or current output, and subsequent design optimization. Figure 11a,c indicate that the flux density close to the internal layer of Halbach array is relatively larger than that near the middle magnet layer. Similarly, Figure 11e,g indicate that the flux density close to the external layer of Halbach array is relatively larger than that near the middle layer. This result is consistent with the comparison in Figure 9. Figure 11 also shows that both radial and axial components of magnetic flux density change alternatively with positive and negative signs, which is caused by the alternatively magnetized PM poles in the axial direction.

## 6. Discussion

The objective of this study is to propose one novel tubular linear machine with hybrid magnet arrays and multiple layers of windings. It could be implemented for actuation or motion sensing depending on particular tasks. The employment of hybrid magnet array with Halbach and alternatively magnetized radial poles helps to enhance the magnetic flux density in the radial direction, and thus increase the current signal strength or force output. Furthermore, the utilization of multiple windings offers the redundancy property, and thus increases the system reliability significantly. Based on the proposed topological design, the magnetic vector potential is formulated analytically from governing equations. The flux distribution is then obtained from the curl of the potential. Numerical computation is conducted to validate the analytical model. It shows that the analytical model agrees with the numerical result well. In addition, the variation of magnetic flux density in the linear machine is consistent with the magnet patterns. The proposed structure topology and developed analytical model could be employed for subsequent study on current signal or force generation, and real-time motion control.

## Figures and Tables

**Table 1:** Comparison of magnetization patterns of linear machines.

Magnet Patterns	Particular Designs	Features
Axial magnetization	Three magnets with axial magnetization	Simple structure, easy control; high flux leakage, low force output
	NS-NS-SN-SN fashion with spacers	High flux density near the like pole regions, improve force output; low volume efficiency
	Convex magnet pole	Low magnetic saturation, low flux leakage; difficult fabrication and assembly
Radial magnetization	Surface-mounted radial magnetization	Less magnet material, high dynamics; lower flux density, special magnetization fixture
	Single ring magnet with multiple sectors	No special requirement of magnetization fixture; high flux leakage, low force output
	Multiple magnets in axial direction	High force output, large working range; magnetization and assembly challenge
	Magnetic screw-nut	Produce force and torque simultaneously; coupled motions in two directions
Halbach array and its extension	Conventional Halbach array	High flux density on one side of PM; flux leakage exists although it is relatively reduced.
	Improved compounded Halbach array	Good shielding effect, low mover mass, high dynamic response; difficult magnet assembly
	T-shaped and trapezoidal magnet	High magnetic flux linkage; Non-regular shape of magnets, difficult for fabrication
	Dual Halbach array	High axial force output, low radial vibration; Force enhancement is not significant, especially for large size machine.

**Table 2:** Major parameters of the linear machine for numerical analysis.

Parameter Items	Value
Thickness of back iron, $R_{1}$	0.005(m)
Outer radius of inner Halbach layer, $R_{2}$	0.010(m)
Inner radius of middle PM layer, $R_{3}$	0.017(m)
Outer radius of middle PM layer, $R_{4}$	0.020(m)
Inner radius of outer Halbach layer, $R_{5}$	0.028(m)
Outer radius of outer Halbach layer, $R_{6}$	0.032(m)
Outer radius of outer back iron, $R_{7}$	0.035(m)
Width of radial magnets, $\tau_{r}$	0.016(m)
Width of axial magnets, $\tau_{z}$	0.008(m)
Pole pitch, $\tau_{p}$	0.024(m)
Permeability of vacuum, $\mu_{0}$	$4\pi \times 10^{-7}\left(N/A^{2}\right)$
Relative permeability of PMs, $\mu_{r}$	$1.07\left(N/A^{2}\right)$
Remenance, $B_{rem}$	1.23(T)
